# A Brief Parenting Intervention to Enhance the Parent–Child Relationship in Hong Kong: Harmony@Home

**DOI:** 10.1007/s10826-012-9614-0

**Published:** 2012-06-24

**Authors:** Cecilia S. Fabrizio, Tai Hing Lam, Malia R. Hirschmann, Sunita M. Stewart

**Affiliations:** 1 School of Public Health, The University of Hong Kong, 5/F William MW Mong Block, Room 5-05, 21 Sassoon Road, Pokfulam, Hong Kong; 2 University of Texas Southwestern Medical Center, Dallas, TX USA

**Keywords:** Parenting, Parent–child relationships, Nonwestern culture, Family, Prevention

## Abstract

There is a dearth of high-level evidence for brief programs designed to promote positive parent–child relationships in nonwestern cultures. We present a pilot randomized controlled trial of a four-session intervention to enhance the parenting skills that promote a positive relationship with pre-adolescent children in Hong Kong. Our intervention, Harmony@Home, utilized Cunningham’s culturally appropriate coping modeling, problem-solving approach to change parental behavior. Our objective was to evaluate the feasibility, acceptability and initial evidence of benefit of the intervention. We blindly randomized 150 Hong Kong parents of children 10–13 years of age to (a) a Harmony@Home intervention group, (b) a waitlist control group, or (c) a third active intervention which shared the control group. Immediately following the intervention, we report increases in satisfaction with the parent–child relationship, one of the targeted parenting behaviors and family harmony, for the Harmony@Home group versus control group. However, only the results from satisfaction with the parent–child relationship were significant at 3-months post intervention. Most respondents reported high levels of program satisfaction. The results provide preliminary evidence that this parenting intervention is culturally acceptable for a nonwestern general population, is feasible for implementation in a community setting and shows evidence of benefit. This intervention is concordant with public health priorities because of the global importance of the parent–child relationship as a protective factor for adolescent outcomes, the need for culturally-appropriate interventions for nonwestern populations, and design characteristics that promote dissemination.

## Introduction

We describe a pilot randomized controlled trial (RCT) of an intervention to enhance parent–child relationships in Hong Kong Chinese families, as a modifiable protective factor for adolescents’ behavioral and academic outcomes. We believe this topic has important social and public health implications internationally for several reasons. First, preventive interventions are particularly important in areas of the world, such as Hong Kong, with a low rate of mental health professionals per capita and strong social stigma that inhibit access to secondary and tertiary care (Ip 2002). Second, while the importance of developing interventions from “within” a culture has been recognized as important (Gergen et al. 1996), such community-based intervention studies from nonwestern cultures are relatively rare. Third, academic-community partnerships are increasingly important to achieving sustainable change, particularly in cultures where community practitioners are not trained in evidence-based approaches, and yet community agencies can offer access to a “healthy” population for preventive interventions. Finally, in applying the concept of the parent–child relationship as a modifiable protective factor to a general population, this study is at the interface of public health and psychology (Lim et al. 2005; Spijkers et al. 2010; Spoth et al. 1998).

Evidence indicates that a positive parent–child relationship in Chinese culture is a modifiable protective factor that can influence behavioral and academic outcomes for adolescents, as well as overall family harmony, a component of family functioning (Chang et al. 2003; Chen et al. 1997; Wong et al. 2007). Although the body of research demonstrating that the specific parenting traits of warmth and autonomy granting can influence the parent–child relationship has been developed primarily in the West (Baumrind 1971; Branstetter and Furman 2012; Steinberg 2001; Steinberg et al. 1989), there is growing evidence that these parental traits mediate similar outcomes in nonwestern, specifically Chinese cultures (Chang et al. 2003; Chen et al. 1997). In addition, those parents that exhibit lower levels of warmth and higher levels of control are more likely to experience increased parent–child conflict and reduced perceptions of family harmony, a salient outcome in Chinese culture (Lau et al. 1990).

There are few RCT reports of interventions developed outside the West to change parental behavior and enhance the parent–child relationship. Western-derived interventions often fail to address local risk factors or utilize culturally inappropriate techniques, such as praising a child’s behavior or encouraging personal assertiveness in group settings (Lau et al. 2011). To be cost-effective and widely accepted, research should be conducted with sensitivity to cultural values, be participatory, build on a preventive science base and include community involvement at all stages (Kumpfer and Alvarado 2003).

Our aim for this study was to test the effectiveness of the intervention in enhancing the parental skills that promote a positive relationship with pre-adolescent children in Hong Kong. Our study hypothesis was that Harmony@Home group participants receiving training in specific parenting skills would report greater increases in parental satisfaction with the parent–child relationship, from pre to post intervention, in comparison to control group participants, and that these effects would be maintained until 3-months follow-up. Our secondary hypothesis was that the increased parental satisfaction with the parent–child relationship would enhance family harmony among the Harmony@Home group, from pre to post intervention, in comparison to the control group, and that these effects would be maintained until 3-months follow-up. Given the early stage of the development of this intervention, the general community population, the direct implementation in the community, and the brief nature of the program, the effect sizes were projected to be moderate.

## Methods

### Development of the Intervention

This FAMILY: Harmony@Home intervention was both developed and trialed in the community, using a collaborative approach between a social service community partner, the Hong Kong Family Welfare Society (HKFWS), and the School of Public Health at The University of Hong Kong (SPH-HKU). This trial was part of a larger project entitled: “FAMILY: a Jockey Club Initiative for a Harmonious Society” (the “FAMILY Project”) that included a longitudinal family cohort study and social marketing programs (described more completely in Stewart et al. 2012). The FAMILY Project’s overarching goal was to enhance family harmony, health, and happiness in Hong Kong by devising a series of interventions and social marketing programs to be disseminated throughout the territory, as evidence of benefit was developed. Program parameters emphasized minimizing community burden and costs in keeping with public health priorities. Features of program design included targeting “healthy” participants to maximize population benefit, brief sessions and use of community-based personnel to minimize costs, and content and delivery methods in accordance with community input to enhance acceptability of social service agencies and community participants.

The process of developing this parenting program within the community-based participatory framework included a community stakeholder needs assessment and discussion groups of potential participants (Stewart et al. 2012). Based upon this input and the preference of the community service partner, we restricted the target group for this preventive intervention to parents with pre-adolescent children. This period is a pivotal time of stress upon the parent–child relationship in Hong Kong, as 12 and 13 year old students take exams that determine whether they qualify for the limited places available in the better secondary schools (Yau and Smetana 1996). Parental anxiety in this academically achievement-oriented culture compels them to increase their control over their children at this time when developmentally the child is seeking greater autonomy.

### Participants

We invited the general community population of Chinese parents with a child aged 10–13 years old, living in the Tseung Kwan O district of Hong Kong (a lower to middle income area with both government-subsidized and private housing) to join this study during January to April 2009. The age criteria were determined by the funding agency, so as to not overlap with other pilot studies independently developed under the FAMILY Project umbrella study (Stewart et al. 2012). Eligibility criteria was quite broad to maximize population reach, as eventually a general community population target will help increase population rates of competent child behavior and decrease rates of problem behavior for maximum public health benefit (Spoth et al. 2002). We required eligible parents to be able to read and write in Chinese at a primary school level, in order to complete the assessments. Parents were deemed ineligible if either they or their child had a serious psychological or behavioral disorder, as determined by self-report.

### Procedures

Our study was intended to encourage strong retention and feasibility and to maximize the public health impact. Therefore we designed in elements that encouraged these goals when feasible, such as a relatively short program (four, 2-hour sessions), and the use of community-based interventionists with brief training.

We recruited a total of 181 potential participants via district schools (invitation letters, pamphlets and informational sessions), the general public (newsletters, road shows, and minibus and newspaper ads) and personal referrals through the social service agency. After initial verbal consent over the telephone, a licensed social worker screened potential participants for eligibility. Sixteen potential participants did not meet the inclusion criteria for the study, seven were not available, and two were originally assigned to groups and began the intervention but were subsequently determined to be ineligible and discontinued from the analysis. We obtained written consent from all participants before the start of the intervention and ethical approval from the Institutional Review Board of the University of Hong Kong/Hospital Authority Hong Kong West Cluster. We delivered the intervention and assessments in Cantonese, the primary local dialect. For the assessments, we used pencil and paper, and participants usually completed them within 20–30 minutes. To enhance retention, we administered assessments at the community agency and compensated study participants HK$200 (about US$30) for completion of the three assessments.

To enhance attendance, we offered childcare arrangements and held the intervention sessions at the community agency in an area of Tseung Kwan O, which was easily accessible by public transportation (Spoth et al. 2002). Each intervention group included eight to twelve participants. If a participant was absent, the facilitator arranged a brief telephone make-up (15–20 minutes) before the next session. For cost-efficiency and sustainability, the facilitators were paraprofessionals, primarily native Cantonese-speaking, licensed social workers from the partner community agency. The Principal Investigator and the agency’s project leader jointly conducted training, which consisted of a 2-day interactive workshop, followed by supervised trial sessions. To maximize consistency of the intervention, sessions were manualized and facilitators were trained to follow the manual closely. Once the study began, facilitators completed a self-assessment after each intervention session to monitor their own adherence to the manual. For fidelity purposes, we videotaped all the sessions (with participant approval) and had third-party trained observers rate the facilitators, using a checklist of the major points to be covered during the session and scale to rank the consistency of the intervention delivery method. We reviewed these fidelity assessments in a weekly meeting with the facilitators and other research team members.

### Study Design

We tested the Harmony@Home intervention as part of a three-group RCT and offered the control group the intervention after the study was completed. A second experimental group received a conflict management intervention, and was tested against the same control for efficiency’s sake. Findings from this intervention will be reported elsewhere, as the objectives and outcomes differed in theory, content, delivery techniques and outcomes. Our study aimed to recruit 150 participants, or 50 per study group, based on an a priori sample size calculation that this number would detect moderate differences (Cohen’s *f* of 0.25), after allowing for a modest dropout (Cohen 1977). Participants were assessed by self-report at pre intervention (T1), post intervention (T2), and 3-months post intervention (T3).

### Intervention

**Fig. 1:**
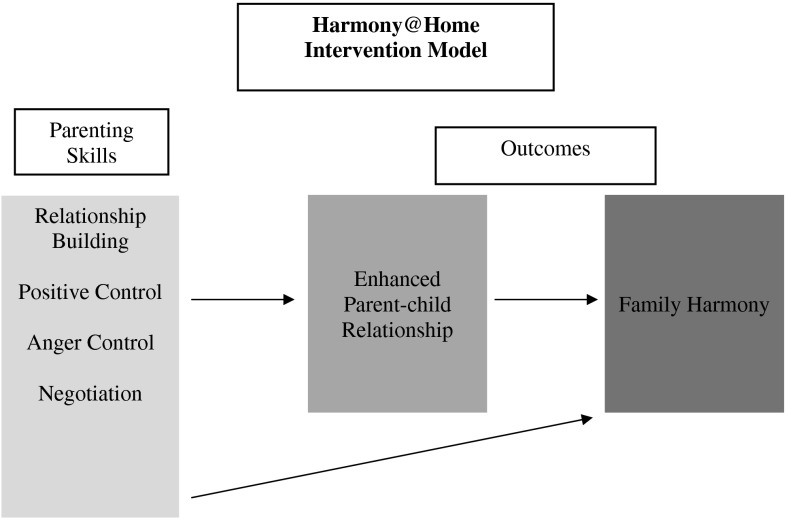
Intervention model

Given the dearth of appropriate evidence-based parenting interventions that have been developed and tested in Chinese cultures, our research team developed a positive preventive program that focused on encouraging parents to increase their warmth toward their children and to decrease their negative control. We kept the intervention brief to encourage attendance, with each of the four 2-hour weekly sessions focused on one of the following parental skills: relationship-building; disciplining misbehavior in a positive manner; controlling anger; and negotiating good behavior (Fig. 1). We targeted these skills to the specific needs identified during the parent discussion groups conducted in the early stage of intervention development, when parents indicated that they did not know how to manage their children without using negative control mechanisms (see Stewart et al. 2012, for more details).

Our research team utilized two key strategies to ensure that the intervention was culturally relevant and acceptable. First, we targeted common behaviors expressed by potential participants in the needs assessments. Next, we designed an intervention that utilized a delivery method adapted from Cunningham et al. (1993) and Cunningham’s (2006) coping modeling, problem solving model. This model was based upon the social learning principle, which employed scenarios with common parent–child interactions to demonstrate parenting “errors”. We then scripted these scenarios and the intervention facilitators acted them out, so that the parents could generate alternative responses using the sessions’ lessons. Because this delivery approach allowed solutions to be generated by the parents themselves, this method was more likely to be respectful of the cultural context than solutions provided by an expert with a theoretical knowledge base but limited experience with the cultural context.

The intervention also drew upon the health action process approach (HAPA) model for behavior change (Schwarzer 2008). This model emphasizes the distinction between the pre-intentional motivation process that drives a person’s behavioral intention and a post-intentional violition process that facilitates the adoption and longer-term maintenance of the specific behavior change (Schwarzer 2008). From this theoretical basis, we designed the intervention to focus on two essential components of change: *intention* to make the desired change; and *planning*, which drives the change from intention to action, and requires the participants to detail how and when they will utilize the target behavior. There is evidence of this model’s effectiveness in changing physician activity behavior in Chinese groups (Schwarzer et al. 2010). After the parents in the group worked together to suggest alternative strategies for the interventionist acting as a parent in the scenarios, the interventionist guided the participants to discuss the long-term consequences of each strategy on the parent–child relationship to further enhance the participants’ intention to change. Then participants created and role-played their own “script,” in preparation for situations when their child misbehaved, and prepared “homework” assignments to practice the skill, to enhance planning, the second component of change.

### Measures

#### Feasibility and Acceptability

We tracked study retention using the CONSORT flow chart to assess the study’s ability to retain sufficient numbers of participants, and monitored intervention session attendance to assess the community’s acceptance of the intervention. In addition, we assessed overall program satisfaction qualitatively and quantitatively. First, immediately after the final intervention session, we asked all respondents to assess the program overall. The program assessment included five questions, using a five-point Likert scale ranging from *strongly disagree* to *strongly agree*: (1) “How much did you like this program?”, (2) “How useful is this program to you?”, (3) “Are you satisfied with the program?”, (4) “Did the program meet your expectations?”, (5) “Would you recommend this program to your friends and relatives?”. Second, we randomly selected ten of the participants in each intervention session, based on a simple random number generator, to join a post intervention discussion group. In these groups, the group facilitator used a semi-structured discussion guide to ask participants their thoughts on the content and delivery of the intervention, as well as their perceived behavioral outcomes.

### Intervention Effects

#### Satisfaction with the Parent–Child Relationship

In order to assess the primary outcome of the parent’s satisfaction with their child, we adapted a single item from the Kansas Marital Satisfaction scale (Schumm et al. 1983). We gave participants a six-point Likert scale to rate the level of satisfaction with their relationship with their child, ranging from *extremely satisfied* to *extremely unsatisfied*.

#### Targeted Behaviors

We measured outcomes with simple, single item questions for the range of potential behaviors targeted in the intervention, as pre-pilot trial groups indicated that these type of questions were more likely to show change than were broader scales of warm or harsh parenting. These types of questions are commonly used in HAPA-based behavior change programs (e.g., Luczynska 2006).

#### Self-Reported Frequency

To measure behavior change, we asked participants to report the frequency of each key parental behavior item they practiced (“How often in the last 2 weeks did you tell your child what to do without repeating yourself over and over?”) with a five-point Likert scale ranging from *never* to *always*.

#### Perceived Change

We supplemented the assessments of behavior change frequency with participants’ subjective assessment of change to maximize capture of the small movement that would be expected following a brief program. To do this, we asked participants to report their perception of the change in the frequency of each key parental behavior item (“Compared to the time before I joined the program, I told my child what to do without repeating myself over and over?”) with a seven-point Likert scale ranging from *decreased a lot* to *increased a lot*.

#### Harmony

In light of its importance in Chinese culture, we also assessed harmony as a secondary outcome. Harmony was measured with an eight-item scale developed by the larger FAMILY Project research team. We asked participants for their level of agreement with each statement regarding their family, such as “my family is harmonious” and “my family functions well for all members,” using a five-point Likert scale ranging from *strongly agree* to *strongly disagree*. We chose this scale after reports from a FAMILY Project cohort of 6,030 general Hong Kong population respondents indicated that all items loaded on a single factor, with Cronbach’s α reflecting good internal consistency of 0.92 and 2-week test–retest reliability of *r* = 0.83 (subset of 467 subjects). Using the same broad sample, the scale also showed evidence of construct validity, as it was positively correlated with the Family APGAR scale for family functioning (*r* = 0.37, *p* < 0.05) (Smilkstein 1978).

## Results

**Fig. 2:**
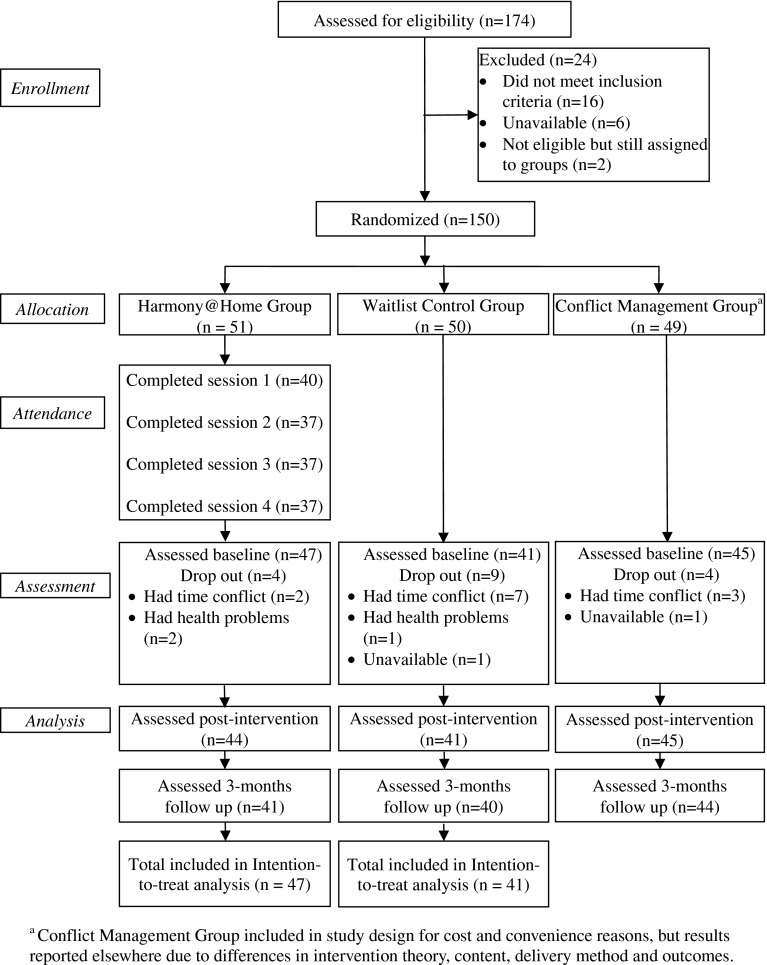
Flow of participants through each stage of the study

We determined that one hundred fifty of the recruited participants were eligible for the study, and were randomly allocated on an individual basis by a trained research assistant using serially numbered, opaque, sealed envelopes (SNOSE) which were prepared using computer-generated random numbers. Of these, we assigned 51 to the Harmony@Home group, 50 to the waitlist control group, and 49 to the other group to be reported elsewhere (see Fig. 2: CONSORT flow statement). Study participants (*n* = 83) had a mean age of 41 years (range 26–57 years) and were 94.1 % female and 5.9 % male. Participants had, on average, two children (mean 1.9, range from 1 to 4). Eighty-four percent of all study participants were married; 59 % worked outside the home, at least part-time; and 74.1 % lived in households earning less than the median annual household income (AHI) for Hong Kong residents (approximately US$29,230; Census and Statistics Department, Hong Kong 2006). Three of the Harmony@Home group participants did not report AHI data.

Based upon independent sample *t* tests and χ^2^ tests, there were no significant differences in age, number of children, place of birth, marital and working status, and household income between the Harmony@Home and control groups at baseline (Table 1).

**Table 1:** Baseline demographic characteristics and baseline outcomes of participants in Harmony@Home Group and control group

Variables	H@H group (*n* = 47)	Control group (*n* = 41)	*p* value^a^
	M (SD) or %	M (SD) or %	
*Demographic characteristics*			
Age^b^	41.32 (5.33)	40.77 (5.91)	0.66
Number of children	1.94 (0.97)	1.90 (0.70)	0.85
Place of birth			0.89
Hong Kong	40.4 %	39.0 %	
Outside Hong Kong	59.6 %	61.0 %	
Marital status			0.77
Married	83.0 %	87.8 %	
Single^c^	17.0 %	12.2 %	
Working status			0.24
Nonworking^d^	51.1 %	63.4 %	
Working	48.9 %	36.6 %	
Household income (HK$)^e^			0.81
<2,000	0.0 %	2.4 %	
2,000–5,999	15.9 %	14.6 %	
6,000–9,999	36.4 %	36.6 %	
10,000–19,000	20.5 %	22.0 %	
20,000–29,000	15.9 %	12.2 %	
30,000–39,000	9.1 %	4.9 %	
>40,000	1.2 %	3.5 %	
Outcomes			
Satisfaction with relationship with child	3.96 (0.96)	3.80 (0.87)	0.44
Family harmony	3.90 (0.70)	3.48 (0.76)	0.01*
Made effort to enhance relationship	3.43 (0.97)	3.10 (0.92)	0.11
Stated clear expectations	4.38 (0.74)	3.83 (0.92)	<0.01*
Gave reasonable consequences	3.40 (0.80)	3.37 (0.99)	0.84
Stayed calm when child argued	2.98 (0.87)	2.95 (1.02)	0.89
Negotiated good behavior	3.89 (0.81)	3.37 (0.80)	<0.01*

^a^ *H*@*H* *group* Harmony@Home group

^b^All *p* values based on independent samples *t* test or χ^2^

^c^ *Sample size* Harmony@Home group, *n* = 44, control group, *n* = 39

^d^Single parent included participants who were never married, divorced or widowed

^e^Nonworking included unemployed and those not working outside of the home

^f^ *Sample size* Harmony@Home group, *n* = 44, control group, *n* = 41

^*^The result is statistically significant at *p* <0.05

Participants assigned to the two groups did not differ on baseline value for the primary outcome of parental satisfaction, although there were baseline differences for two of the targeted behaviors and for family harmony (Table 1).

### Feasibility and Acceptability

The CONSORT statement (Fig. 2) shows that study retention was high, with overall retention at 82 %, and similar levels of retention between the Harmony@Home group (78 %), and the control group (80 %). Attendance was high, with session participation, including make-up, ranging from 78 to 85 % of those randomized to the Harmony@Home group. In addition, 93 % of those who attended at least one intervention session of the Harmony@Home group attended all four sessions.

Participant acceptability of the program was strong, based on quantitative and qualitative results. In an assessment immediately post intervention, parents in the experiential group reported strong positive reactions. One hundred percent of respondents ranked the program at least four out of five potential points for “liked the program” and “program was useful” (including “liked” and “liked very much,” or “useful” and “very useful”). Ratings were similar for the other three affective assessments: for “satisfaction with the program” (97 % of participants ranked the program at least four out of five potential points); “meeting their expectations” (89 % ranked the program at least four out of five potential points); and for their “likelihood of recommending it to friends or a relative” (86 % ranked the program at least four out of five potential points). These data were supplemented with qualitative data from post intervention discussion groups. In these discussion groups, many participants noted how this program was better than other parenting programs they had experienced, such as this participant’s comment:

Other programs just teach you how to do it. In this program you have to think of the strategies by yourself. We discussed the methods together. There was lots of self-reflection when we discussed … and which parenting method was best to use … It gave me a deep impression. With previous programs, I’d forget everything when I was home.

(Participant in Harmony@Home group)

Other parents commented on the change in their relationship with their child, such as this participant’s comment:

When I practised the strategies at home I saw changes. So I tried to negotiate and talk with him and use a softer voice and it’s really quite effective. Therefore it’s different from what I thought in the past after listening to the talks, that why should I compromise when I was so exhausted after work? Yet after a few sessions, I tried to change gradually and could see the effect.

(Participant in Harmony@Home group)

### Fidelity

The trained fidelity raters rated 95 % of the Harmony@Home components at full fidelity to the manual. We attributed these strong results to the facilitator’s training, supervision, and the weekly fidelity review meetings that prioritized adherence to the manual.

### Intervention Effects

We utilized repeated measures analysis of covariance (ANCOVA) models to test whether the intervention group reported greater changes in the expected direction for the study outcomes, pre to post intervention and from pre intervention to 3-months post intervention (group was a two-level between-subjects factor; time was a two-level within-subjects factor). Cohen’s (1977) effect size index *f* (0.10 = small, 25 = medium, and 0.40 = large) was used as the effect size estimator for the group by time interaction. We employed a conservative “intention-to-treat” analysis by imputing data, using the method of “last observation carried forward.” Results were similar to the analysis using listwise deletion of cases with missing data; therefore we report only intention-to-treat results. Those who did not complete the baseline assessment after randomization were excluded.

#### Parental Satisfaction with Relationship with Child

Table 2 shows that the intervention was effective for the primary outcome of increasing parental satisfaction with their relationship with their child pre to post intervention for the Harmony@Home group versus control group [*F*(1, 85) = 5.54, *p* = 0.02]. This benefit persisted at 3-months post intervention but was only marginally significant [*F*(1, 85) = 2.77, *p* = 0.10].

**Table 2:** Mean changes in study outcomes, ANCOVA, with respective baseline score as covariate

Outcomes	Δ H@H group (*n* = 47)	Δ Control group (*n* = 41)	*F* statistic	*p* value	ES^a^
Satisfaction with relationship with child					
Pre to post intervention	0.30	0.05	5.54	0.02*	0.25
Pre to 3-months post intervention	0.26	0.07	2.77	0.10	0.18
Targeted behaviors: self-reported frequencies					
Made effort to enhance relationship					
Pre to post-intervention	−0.15	0.05	0.04	0.85	0.00
Pre to 3-months post-intervention	−0.02	0.02	0.63	0.43	0.08
Stated clear expectations					
Pre to post-intervention	−0.32	−0.07	0.11	0.74	0.03
Pre to 3-months post-intervention	−0.43	−0.05	0.41	0.53	0.07
Gave reasonable consequences					
Pre to post-intervention	−0.38	−0.22	0.89	0.35	0.10
Pre to 3-months post-intervention	−0.38	−0.44	0.40	0.53	0.07
Stayed calm when child argued					
Pre to post-intervention	0.32	0.07	3.17	0.08	0.19
Pre to 3-months post-intervention	0.19	0.07	0.97	0.33	0.11
Negotiated good behavior					
Pre to post-intervention	−0.32	−0.32	8.71	0.04*	0.32
Pre to 3-months post-intervention	−0.32	0.02	0.21	0.65	0.04
Family harmony					
Pre to post intervention	0.14	0.02	9.39	<0.01*	0.33
Pre to 3-months post intervention	0.06	0.28	0.58	0.45	0.08

Positive change scores indicate an increase and negative change scores indicate a decrease in study outcomes

*H*@*H group* Harmony@Home group

* The result is statistically significant at *p* < 0.05

^a^ *ES* Cohen’s effect size index *f*: 0.10 = small, 0.25 = medium, 0.40 = large

#### Target Behaviors—Self-Reported Frequency

The Harmony@Home group reported a significantly higher frequency of the target behaviors for one of the five behaviors (“negotiate good behavior”), pre to post intervention, versus the control group [*F*(1, 85) = 8.71, *p* = 0.04] (Table 2). Effect sizes were medium. However, the differences became non-significant at 3-months post intervention.

#### Target Behaviors—Perceived Change

Pre to post intervention, the Harmony@Home group reported significantly greater change compared to the control group for all five target behaviors, three of which remained significant at 3-months post intervention (Table 3). Effect sizes ranged from small to large.

**Table 3:** Means for behavioral study outcomes, one-way ANOVA

Targeted behaviors: perceived change	H@H group (*n* = 44)	Control group (*n* = 41)	*F* statistic	*p* value	ES^a^
	M (SD)	M (SD)			
Made effort to enhance relationship					
Post intervention	3.05 (0.91)	2.37 (0.97)	11.08	<0.01*	0.37
3-months post intervention	3.11 (0.78)	2.46 (0.75)	15.32	<0.01*	0.43
Stated clear expectations					
Post intervention	2.61 (0.99)	2.07 (1.06)	5.90	0.02*	0.27
3-months post intervention	2.70 (1.00)	2.02 (0.94)	10.73	<0.01*	0.36
Gave reasonable consequences					
Post intervention	3.05 (0.94)	2.46 (1.19)	6.34	0.01*	0.28
3-months post intervention	2.98 (0.90)	2.54 (1.03)	4.43	0.04*	0.23
Stayed calm when child argued					
Post intervention	3.02 (0.76)	2.07 (1.08)	22.13	<0.01*	0.52
3-months post intervention	2.98 (1.09)	2.71 (1.03)	1.37	0.15	0.13
Negotiated good behavior					
Post intervention	3.05 (0.89)	2.27 (1.16)	12.10	<0.01*	0.38
3-months post intervention	2.95 (1.10)	2.54 (1.08)	3.14	0.08	0.37

*H*@*H group* Harmony@Home group

* The result is statistically significant at *p* < 0.05

^*a*^ *ES* Cohen’s effect size index *f*: 0.10 = small, 0.25 = medium, 0.40 = large

#### Harmony

The intervention was effective in increasing the level of harmony, pre to post intervention, for the Harmony@Home group versus the control group [*F*(1, 85) = 9.39, *p* <0.01] (Table 2). This difference was not sustained at 3-months post intervention.

## Discussion

We used an RCT design to test a parenting intervention for a general community target, incorporating elements to minimize costs and maximize the public health impact, such as a relatively short program (four, 2-hour sessions), and the use of community-based interventionists with brief training. Interventionists trained parents to enhance their relationship with their child with the use of specific parenting skills that had been prioritized in a needs assessment, and then to practice these skills at home to enhance their efficacy. Cunningham et al.’s (1993) and Cunningham’s (2006) COPE problem-solving model was utilized to maximize the cultural appropriateness for this nonwestern society.

This RCT shows that our parenting intervention was acceptable to the nonwestern target audience and feasible for the community partners to implement. Retention and participation were high, perhaps due to efforts to develop a culturally appropriate intervention, to limit the number of sessions, to offer the program in a convenient venue, and to provide childcare. The community agency found it feasible to execute the recruitment, to train and supervise the interventionists, and to maintain high attendance, which indicates the potential for this program to be sustained after the research is complete.

We found that the Harmony@Home intervention showed evidence of benefit for the primary outcome of improving the parent’s satisfaction with their relationship with their child, and desirable changes in the frequency of the targeted parenting behaviors and perceptions of increased frequency of these behaviors. We also found that the intervention group showed evidence of benefit for enhanced family harmony. However, the post intervention effect sizes were relatively small and only the primary parental satisfaction with the parent–child relationship showed borderline significant benefit at 3-months post intervention. This is consistent with the characteristics of general population, family-focused preventive interventions, as the general population sample is often heterogeneous for the targeted outcomes and may be reluctant to attend more than a brief program for preventive purposes (Spoth et al. 1998). In addition, responses in the Harmony@Home group might have been suppressed post intervention by a greater understanding of the assessment terminology (“clear expectations” or “negotiate”) due to the intervention’s effect on clarifying the assessment terms, while the control group was not influenced by the intervention’s content. Importantly for this pilot stage of intervention development, in quantitative and qualitative feedback that assessed affective response, utility and willingness to recommend the program to others, most Harmony@Home group participants reported strong positive responses to the intervention.

There were some limitations to the study. The sample was relatively small in size, which may have limited its power to detect statistically significant differences for some outcomes. In addition, there was some inconsistency in results between the two measures of the target parenting behaviors, as the measure of perceptions of increased frequency of these behaviors showed more sensitivity to change than the measure of the reported frequency of the targeted parenting behaviors. This difference may be subject to social desirability bias. Finally, follow-up was relatively brief for this pilot study, as many family interventions, some with more intensive programs, continue follow-up for at least 1 year (Taylor and Biglan 1998).

During the next phase of the study, we will focus the program content and associated assessment questions more on the desired behavior change, as the pilot studies’ post intervention gains in behavior-specific frequency were not substantial. We identified measurement issues as the participants readily reported positive change, but the scales used did not capture these changes effectively. Based on the post intervention qualitative feedback, the specific terminology the research participants used appears to be important and may better capture behavioral changes. Therefore we will word the desired skills more precisely in the scripted sessions and in the at-home practice workbooks. For example, instead of “stating clear expectations,” parents will be encouraged to reduce the frequency of “repeating themselves over and over.”

We also observed during the intervention that many of the participants, raised traditionally in homes where parents demanded unquestioning obedience, needed time to discuss issues such as “spoiling” children during the session on negotiating good behavior. Therefore, in future intervention studies we will guide the facilitator to expend more time on attributional questions about the parental behavior’s long-term impact on the parent–child relationship and subsequently on family harmony, to increase participant motivation to use the target behavior. Importantly we will retain the program elements that aim to maximize reach and sustainability, such as the general community target, the program’s brevity, and the use of community facilitators, to maximize its potential public health impact.

## Conclusion

There is strong evidence that behavioral family interventions can be protective for adolescent problem behaviors later in adolescence and adulthood (Stormshak et al. 2009; Taylor and Biglan 1998; Zubrick et al. 2005). Spoth et al. (2002) emphasize the potential for greater public health impact with family-oriented intervention studies that are both scientifically rigorous (such as RCTs) and designed with elements that enable scale-up to maximize reach (such as a general community target and low cost components).

Most family-oriented interventions targeted to a Chinese population were developed in the West, were designed for immigrant populations, or were too burdensome for healthy general populations (Dumas et al. 1999; Hong et al. 2011; Leung et al. 2003). Our study adds to the literature on Chinese parenting by showing preliminary evidence of benefit for a brief, culturally appropriate, general population, parenting intervention. These RCT pilot results suggest that this intervention has the potential for broad application to enhance the protective benefit of the parent–child relationship. In a Chinese society that values harmony, these results also provide support for an intervention to enhance family harmony. We are pursuing a main study that will address the study’s limitations and improve behavior-specific outcomes, while retaining the program elements that contribute to its potential reach and sustainability, if subsequent results justify broader dissemination.

## References

[CR1] Baumrind D (1971). Current patterns of parental authority. Developmental Psychology Monograph.

[CR2] Branstetter, S. A., & Furman, W. (2012). Buffering effect of parental monitoring knowledge and parent–adolescent relationships on consequences of adolescent substance use. *Journal of Child and Family Studies* (published online February 24, 2012). 10.1007/s10826-012-9568-2 PMC3711550 23869161

[CR3] Census and Statistics Department. (2006). *Thematic report*: *Household income distribution in Hong Kong.* Retrieved May 11, 2012, from http://www.legco.gov.hk/yr04-05/chinese/hc/sub_com/hs51/papers/hs510710-rpt070618-ec.pdf.

[CR4] Chang L, Schwartz D, Dodge KA, McBride-Chang C (2003). Harsh parenting in relation to child emotion regulation and aggression. Journal of Family Psychology.

[CR5] Chen X, Dong Q, Zhou H (1997). Authoritative and authoritarian parenting practices and social and school performance in Chinese children. International Journal of Behavioural Development.

[CR6] Cohen, J. (1977). *Statistical power analysis for the behavioral sciences* (revised edition). New York: Academic Press.

[CR7] Cunningham CE, Barkley R (2006). Chapter 13: COPE. Large-group, community-based, family-centered parent training. Attention-deficit hyperactivity disorder. A handbook for diagnosis and treatment.

[CR8] Cunningham CE, Davis JR, Bremner R, Rzasa T, Dunn K (1993). Coping modeling problem-solving versus mastery modeling: Effects on adherence, in session process and skill acquisition in a residential parent training program. Journal of Consulting and Clinical Psychology.

[CR9] Dumas JE, Rollock D, Prinz RJ, Hops H, Blechman EA (1999). Cultural sensitivity: Problems and solutions in applied and preventive intervention. Applied and Preventive Psychology.

[CR10] Gergen KJ, Gulerce A, Lock A, Misra G (1996). Psychological science in cultural context. American Psychologist.

[CR11] Hong L, Wang Y, Agho K, Jacobs J (2011). Preventing behavior problems among elementary schoolchildren: Impact of a universal school-based program in China. Journal of School Health.

[CR32] Ip YM (2002). Mental health promotion in Hong Kong. Hong Kong Journal of Psychiatry.

[CR12] Kumpfer KL, Alvarado R (2003). Family-strengthening approaches for the prevention of youth problem behaviors. American Psychologist.

[CR13] Lau S, Lew WJF, Hau KT, Cheung PC, Berndt TJ (1990). Relations among perceived parental control, warmth, indulgence, and the family harmony of Chinese in mainland China. Developmental Psychology.

[CR14] Lau AS, Fung JJ, Ho LY, Liu LL, Gudiño OG (2011). Parent training with high-risk immigrant Chinese families: A pilot group randomized trial yielding practice-based evidence. Behavior Therapy.

[CR15] Leung C, Sanders MR, Leung S, Mak R, Lau J (2003). An outcome evaluation of the implementation of the triple p-positive parenting program in Hong Kong. Family Process.

[CR16] Lim M, Stormshak EA, Dishion TJ (2005). A one-session intervention for parents of young adolescents: Videotape modeling and motivational group discussion. Journal of Emotional and Behvioral Disorders.

[CR17] Luczynska A (2006). An implementation intentions intervention, the use of a planning strategy, and physical activity after myocardial infarction. Social Science and Medicine.

[CR18] Schumm WR, Nichols CW, Shectman KL, Grigsby CC (1983). Characteristics of responses to the Kansas marital satisfaction Scale by a sample of 84 married mothers. Psychological Reports.

[CR19] Schwarzer R (2008). Modeling health behavior change: How to predict and modify the adoption and maintenance of health behaviors?. Applied Psychology.

[CR20] Schwarzer R, Cao DS, Lippke S (2010). Stage-matched minimal intervention to enhance physical activity in Chinese adolescents. Journal of Adolescent Health.

[CR21] Smilkstein G (1978). The family APGAR: A proposal for a family function test and its use by physicians. Journal of Family Practice.

[CR22] Spijkers W, Jansen DC, de Meer G, Reijneveld SA (2010). Effectiveness of a parenting programme in a public health setting: A randomised controlled trial of the positive parenting programme (triple P) level 3 versus care as usual provided by the preventive child healthcare (PCH). BMC Public Health.

[CR23] Spoth R, Redmond C, Shin C (1998). Direct and indirect latent-variable parenting outcomes of two universal family-focused preventive interventions: Extending a public health-oriented research base. Journal of Consulting and Clinical Psychology.

[CR24] Spoth, R. L., Kavanagh, K., & Dishion, T. J. (2002). Family-centered preventive intervention science: Toward benefits to larger populations of children, youth, and families.* Prevention Science, **3*, 145–152. 10.1023/a:1019924615322 12387551

[CR25] Steinberg L (2001). We know some things: Parent–adolescent relationships in retrospect and prospect. Journal of Research on Adolescence.

[CR26] Steinberg L, Elmen JD, Mounts NS (1989). Authoritative parenting, psychosocial maturity, and academic success among adolescents. Child Development.

[CR27] Stewart SM, Fabrizio CS, Hirschmann MR, Lam TH (2012). Developing community-based preventive interventions in Hong Kong: A description of the first phase of the FAMILY project. BMC Public Health.

[CR28] Stormshak EA, Connell A, Dishion TJ (2009). An adaptive approach to family-centered intervention in schools: Linking intervention engagement to academic outcomes in middle and high school. Prevention Science.

[CR29] Taylor TK, Biglan A (1998). Behavioral family interventions for improving child-rearing: A review of the literature for practitioners and policy makers. Clinical Child and Family Psychology Review.

[CR30] Wong Q, Pomerantz EM, Chen H (2007). The role of parents’ control in early adolescents psychological functioning: A longitudinal investigation in the United States and China. Child Development.

[CR31] Yau J, Smetana JG (1996). Adolescent–parent conflict among Chinese adolescents in Hong Kong. Child Development.

[CR33] Zubrick SR, Ward KA, Silburn SR, Lawrence D, Williams AA, Blair E (2005). Prevention of child behavior problems through universal implementation of a group behavioral family intervention. Prevention Science.

